# Supplementary figures and images for: Selective dephosphorylation by PP2A-B55 directs the meiosis I-meiosis II transition in oocytes
Source: eLife. 2021 Aug 3;10:e70588. doi: 10.7554/eLife.70588 (PMC8370769; doi:10.7554/eLife.70588)

Figure 2-S2-source data

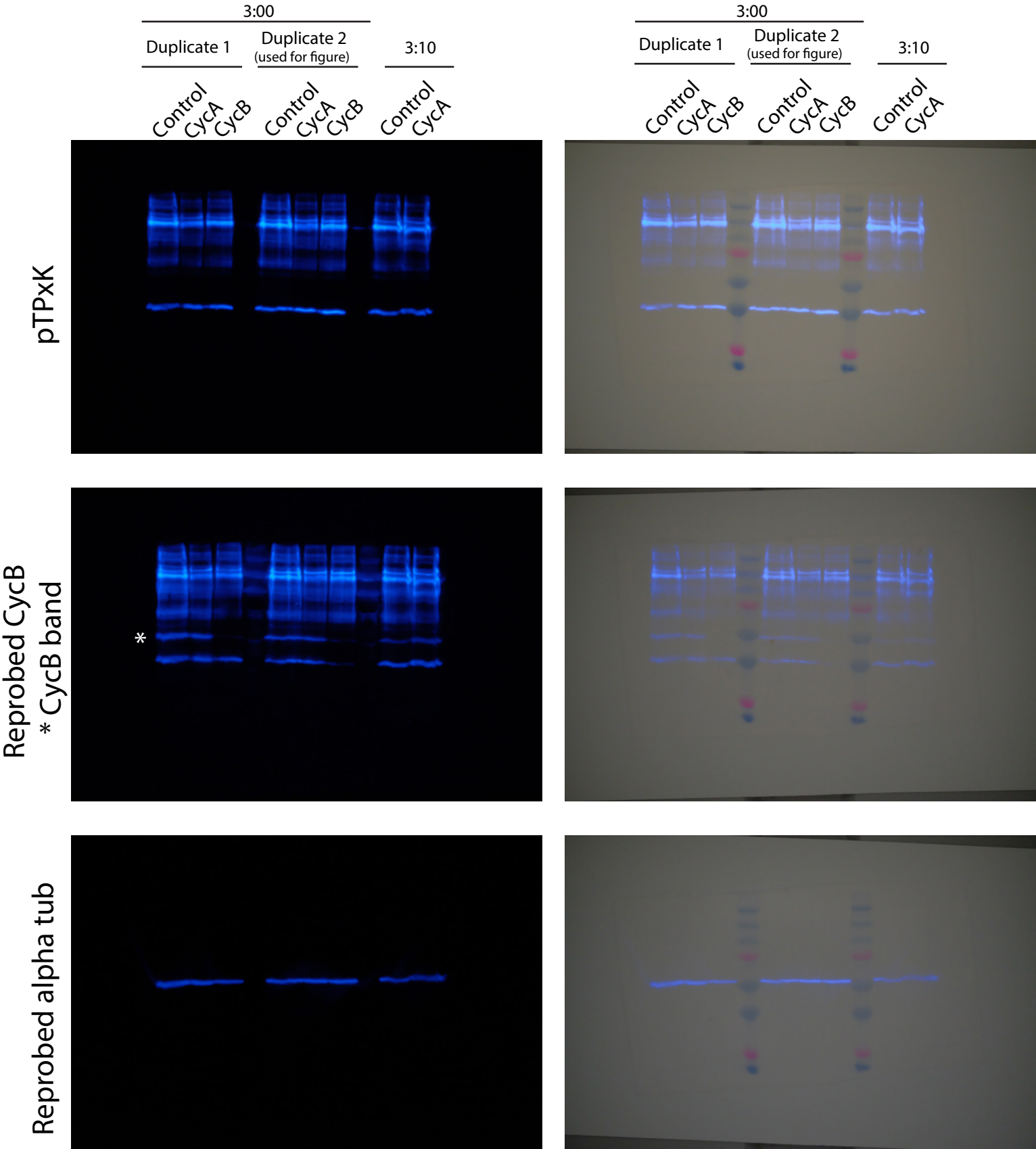

Supplement: Figure 2—figure supplement 2—source data 1. — Western blots of embryos at metaphase of first cleavage (collected at 3 hr and 3 hr 10 min after hormone addition) using antibodies against pTPxK consensus, cyclin B (the specific band indicated with an asterisk), or alpha-tubulin. [file elife-70588-fig2-figsupp2-data1.pdf]

Figure 3 - source data

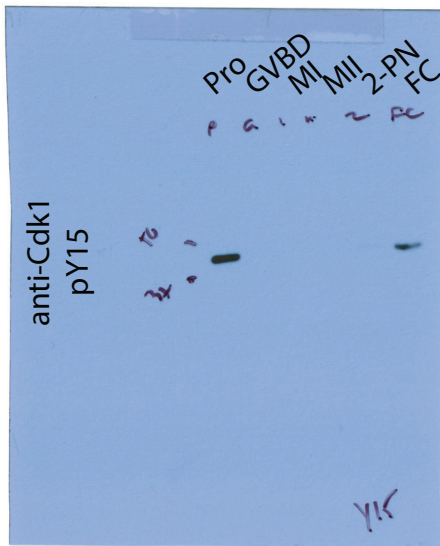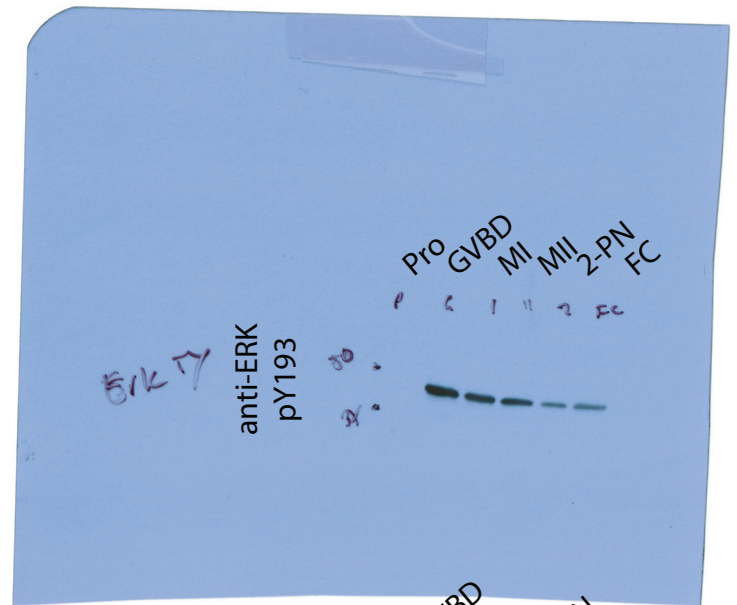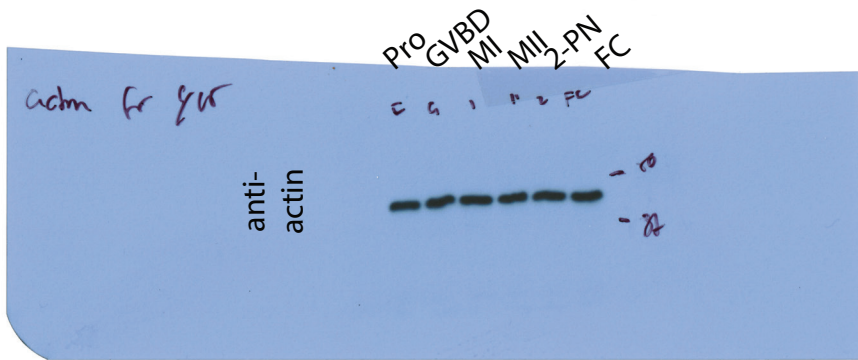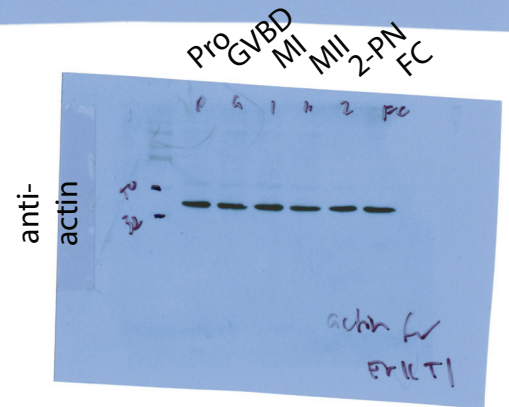

Supplement: Figure 3—source data 1. — Western blots with antibodies recognizing the inhibitory phosphorylation on Cdk1Y15 and activating phosphorylation on ERK Y193, respectively. [file elife-70588-fig3-data1.pdf]

Figure 3-S3-source data

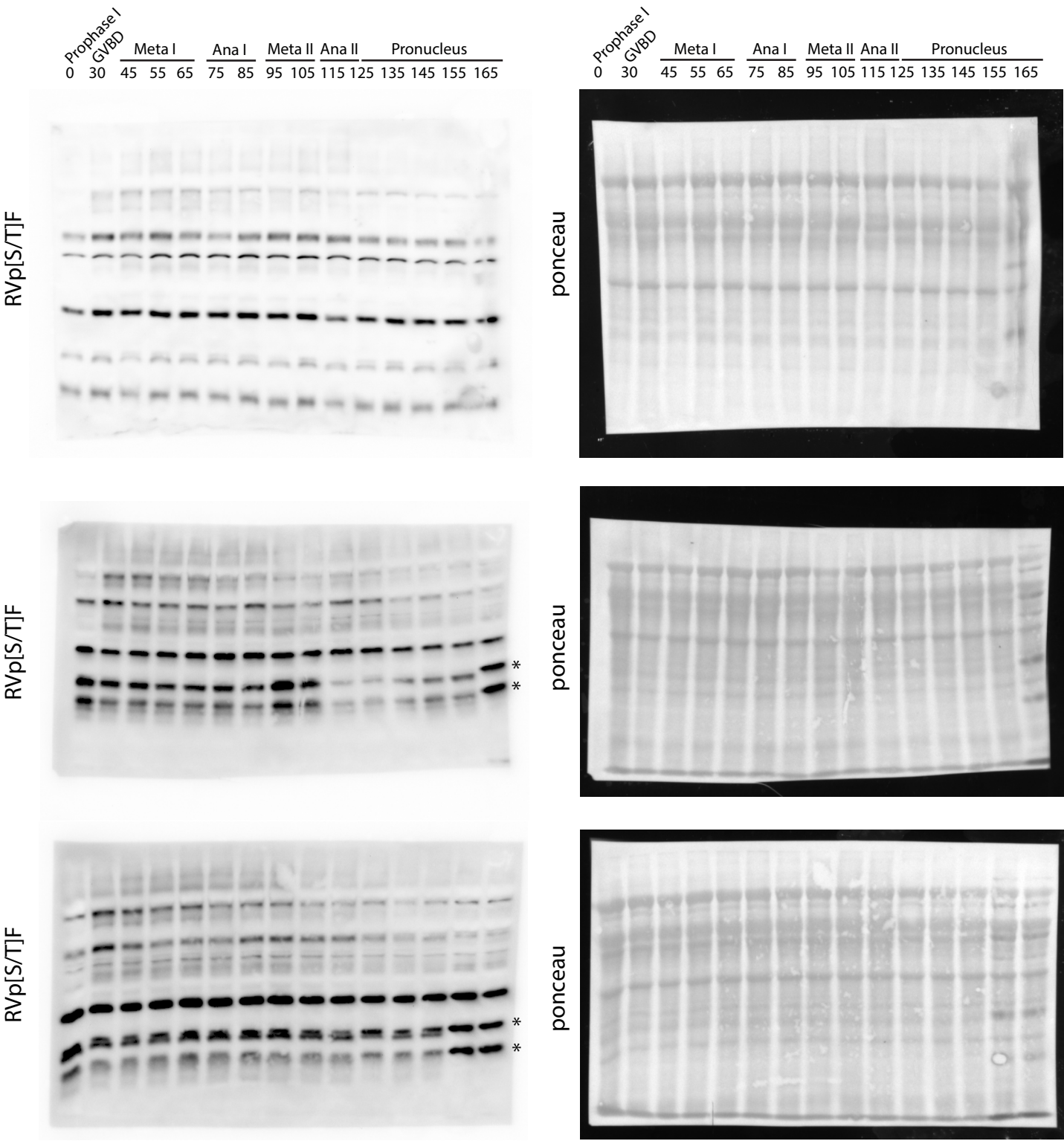

\* indicates protein ladder crossreacted with antibodies

Supplement: Figure 3—figure supplement 3—source data 1. — (A) Time-course western blots of meiotic oocytes using antibodies against RVp[S/T]F motif. Ponceau staining is provided for loading control. [file elife-70588-fig3-figsupp3-data1.pdf]

Figure 3-S4-source data

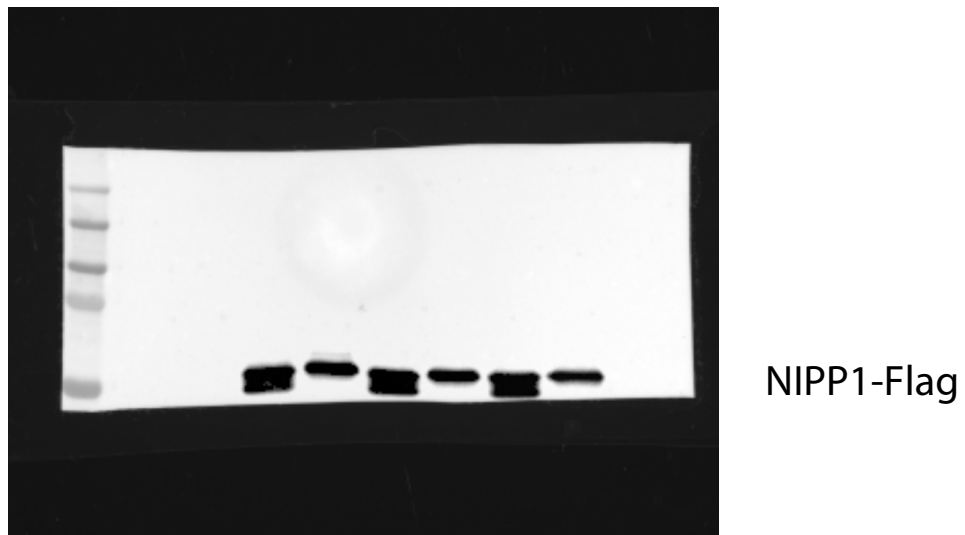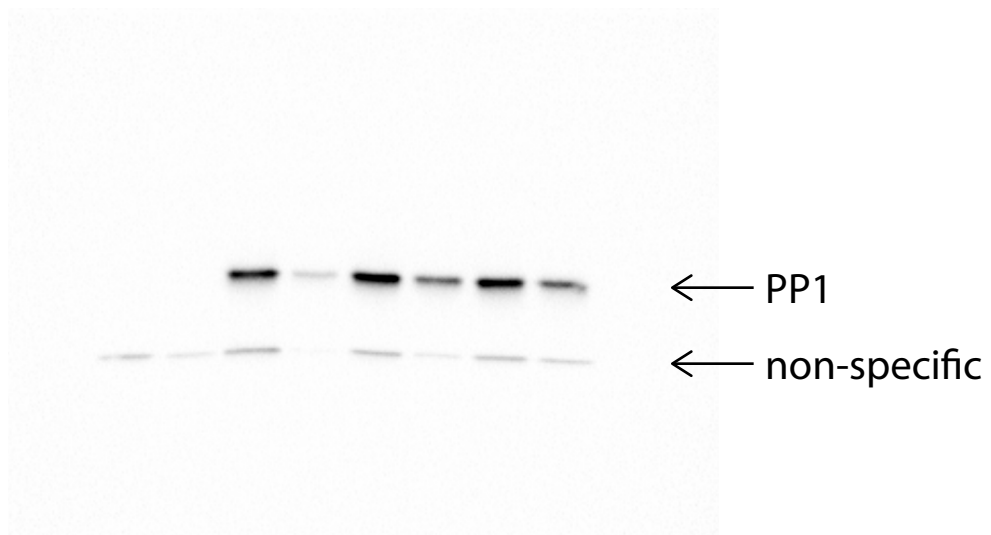

Supplement: Figure 3—figure supplement 4—source data 1. — Immunoprecipitation of wild-type or phosphonull nuclear inhibitor of PP1 (NIPP1) mutants tagged with FLAG, followed by western blot to test PP1 association. [file elife-70588-fig3-figsupp4-data1.pdf]

Figure 3-S5-source data

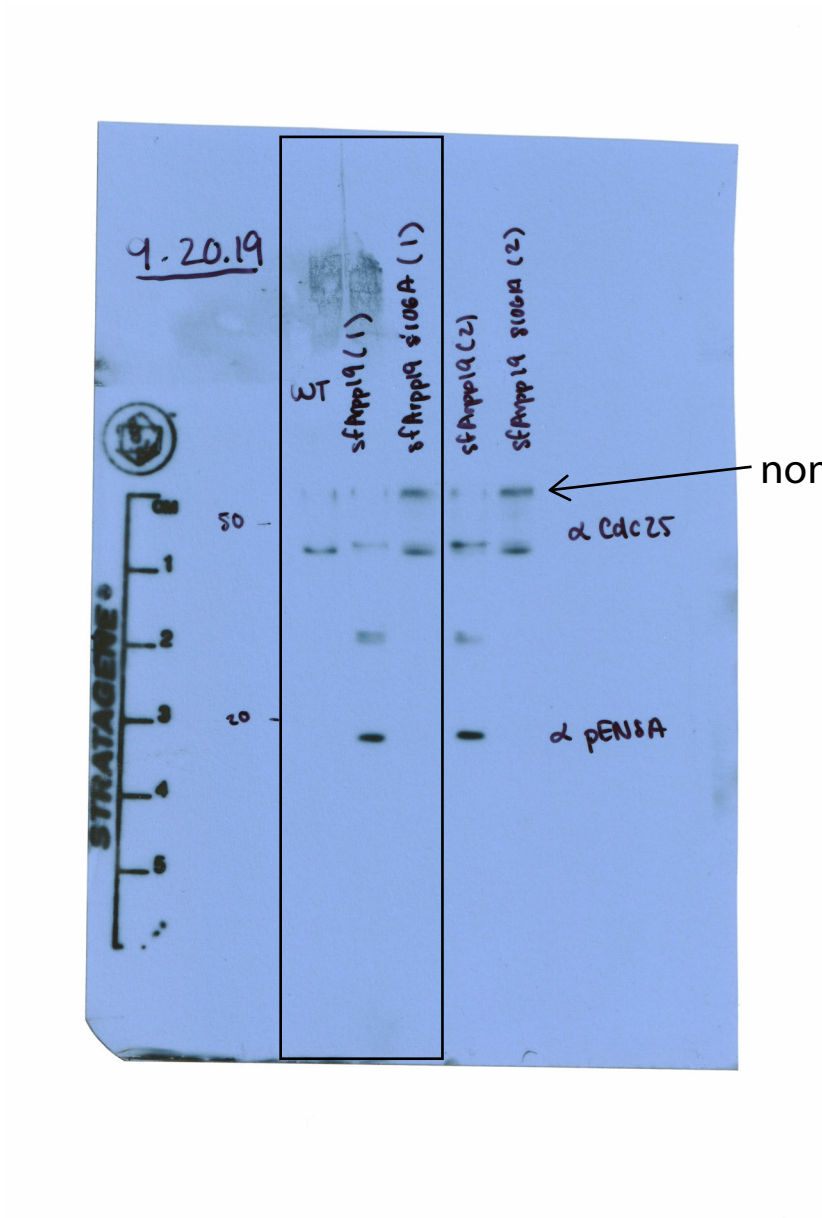

Supplement: Figure 3—figure supplement 5—source data 1. — Western blot of Cdc25 and pENSA in control cell lysate (lane 1) or lysate to which thiophosphorylated PmArpp19WT (lanes 2, 4) or PmArpp19S106A (lanes 3, 5) was added. [file elife-70588-fig3-figsupp5-data1.pdf]

Figure 4-S1-source data

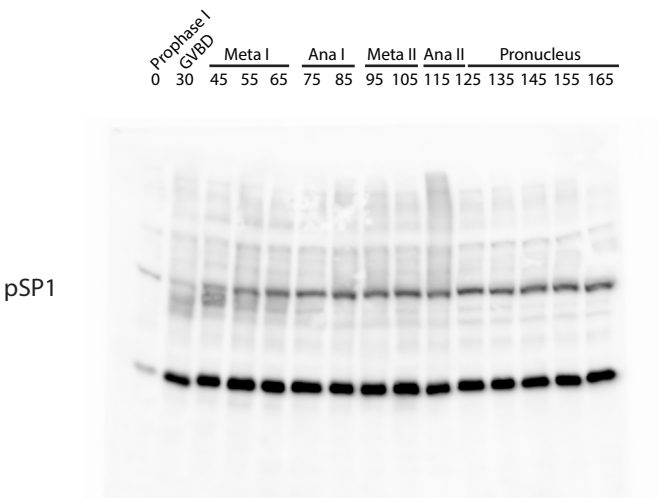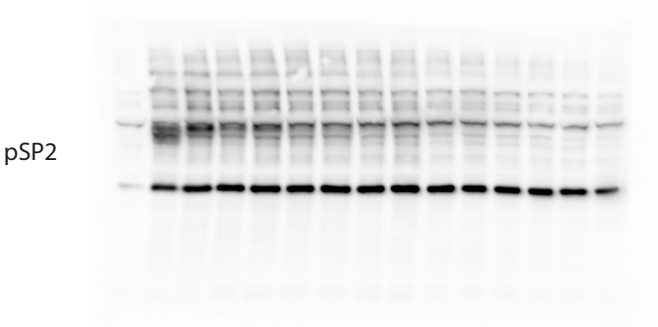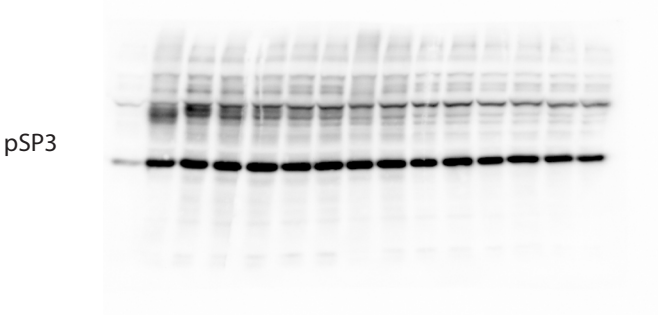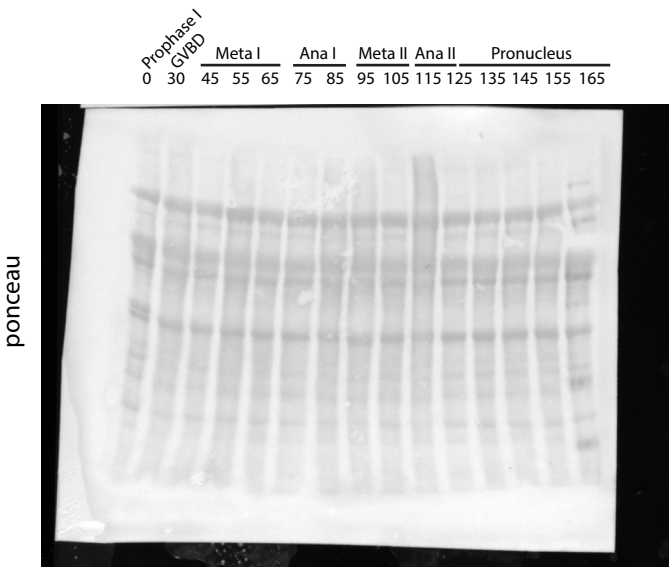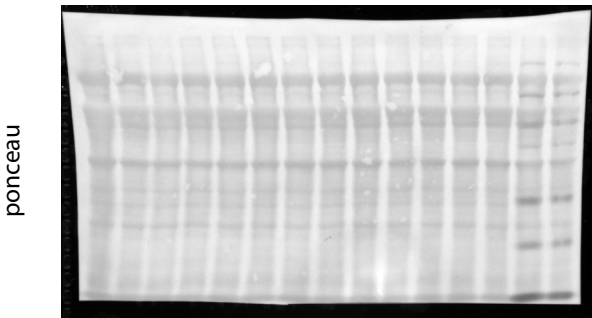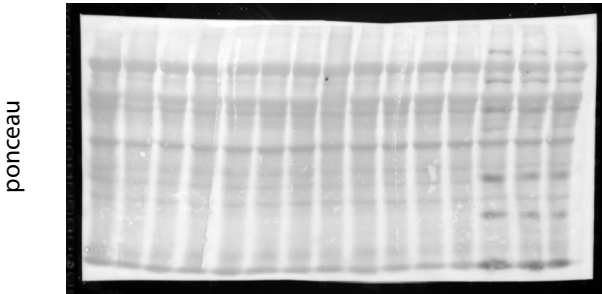

Supplement: Figure 4—figure supplement 1—source data 1. — Time-course western blots of meiotic oocytes using antibodies against (H/K)pSP or pTPxK, respectively. Ponceau staining is provided for loading control. [file elife-70588-fig4-figsupp1-data1.zip › Figure-4-Supplement-1-source-data1.pdf]

Figure 4-S1-source data 2

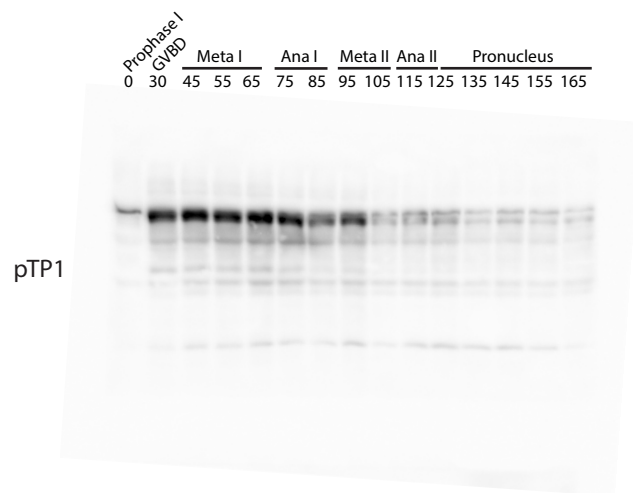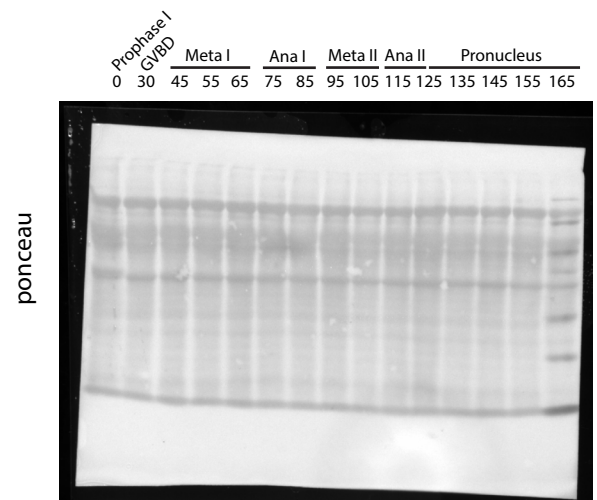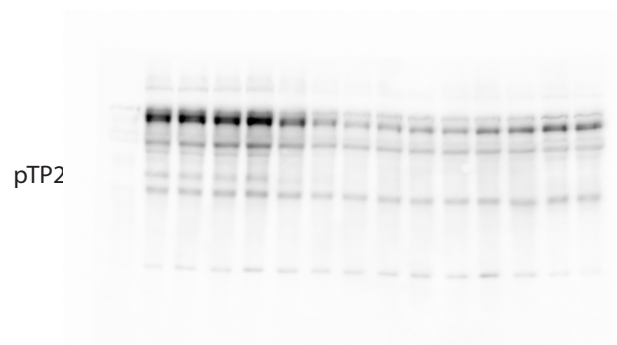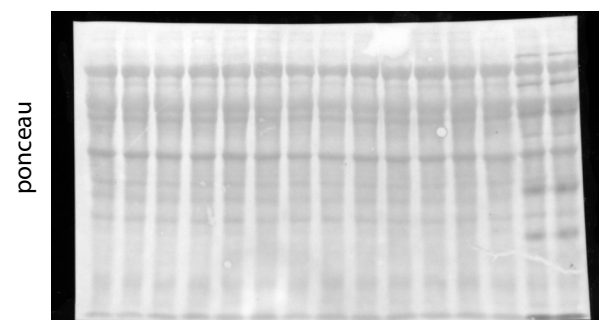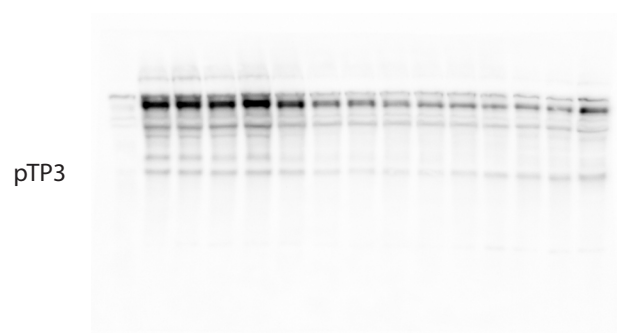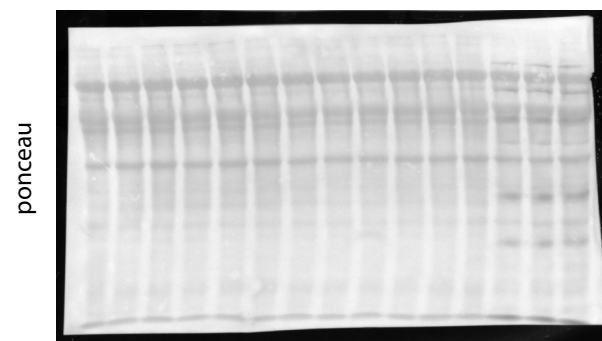

Supplement: Figure 4—figure supplement 1—source data 1. — Time-course western blots of meiotic oocytes using antibodies against (H/K)pSP or pTPxK, respectively. Ponceau staining is provided for loading control. [file elife-70588-fig4-figsupp1-data1.zip › Figure-4-Supplement-1-source-data2.pdf]

Figure 4-S2-source data

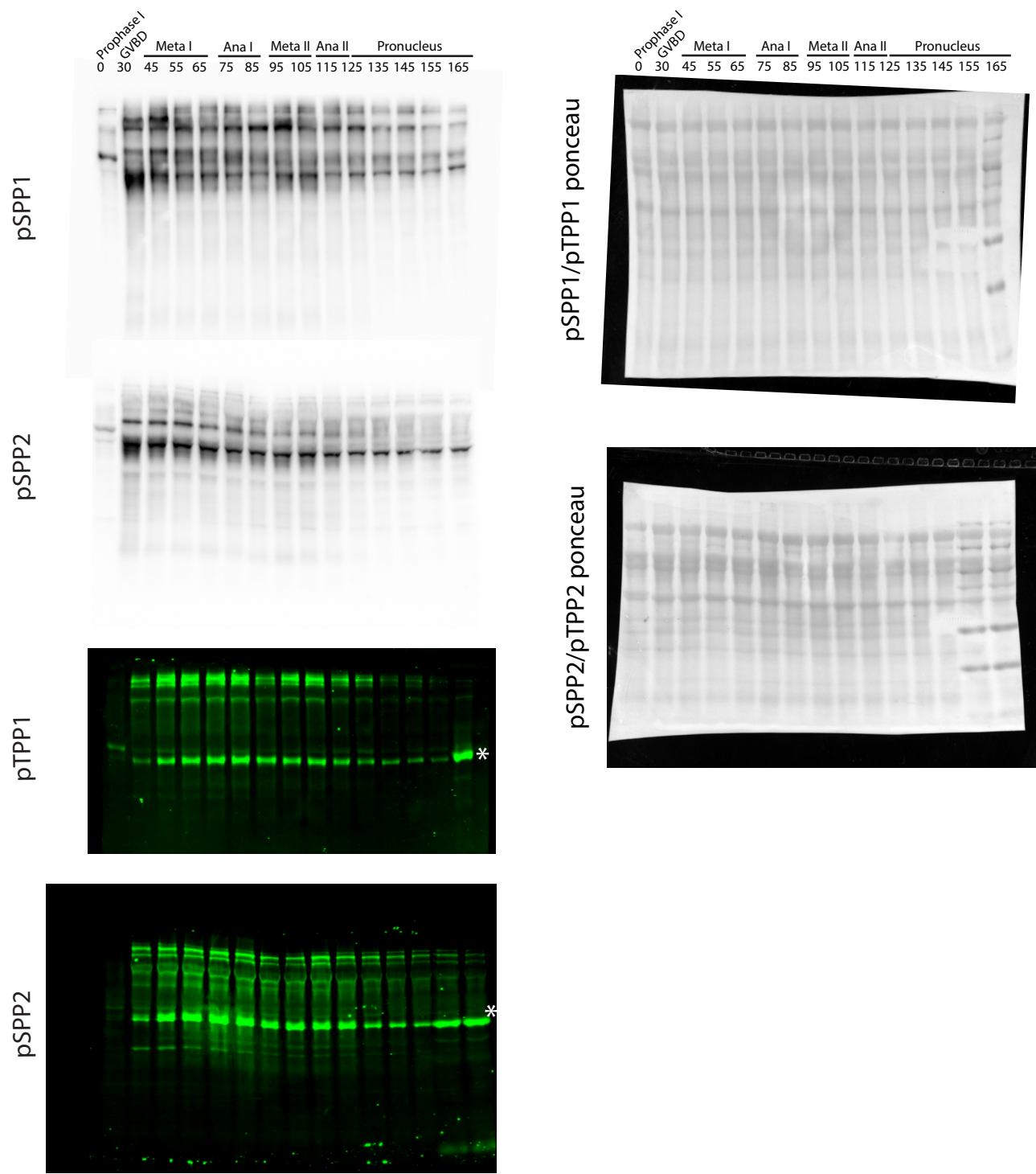

\* indicates protein ladder crossreacted with antibodies

Supplement: Figure 4—figure supplement 2—source data 1. — Time-course western blots of meiotic oocytes using antibodies against pSPP or pTPP. Ponceau staining is provided for loading control. [file elife-70588-fig4-figsupp2-data1.pdf]
